# Optical and Electric Multifunctional CMOS Image Sensors for On-Chip Biosensing Applications

**DOI:** 10.3390/ma4010084

**Published:** 2010-12-29

**Authors:** Takashi Tokuda, Toshihiko Noda, Kiyotaka Sasagawa, Jun Ohta

**Affiliations:** 1 Nara Institute of Science and Technology, 8916-5 Takayama, Ikoma, Nara 630-0101, Japan; E-Mails: t-noda@ms.naist.jp (T.N.); sasagawa@ms.naist.jp (K.S.); ohta@ms.naist.jp (J.O.); 2 PRESTO, Japan Science and Technology Agency, 3–5 Sanba, Chiyoda, Tokyo, 102-0075, Japan

**Keywords:** CMOS image sensor, on-chip imaging, electric imaging

## Abstract

In this review, the concept, design, performance, and a functional demonstration of multifunctional complementary metal-oxide-semiconductor (CMOS) image sensors dedicated to on-chip biosensing applications are described. We developed a sensor architecture that allows flexible configuration of a sensing pixel array consisting of optical and electric sensing pixels, and designed multifunctional CMOS image sensors that can sense light intensity and electric potential or apply a voltage to an on-chip measurement target. We describe the sensors’ architecture on the basis of the type of electric measurement or imaging functionalities.

## 1. Introduction

The complementary metal-oxide-semiconductor (CMOS) image sensor is one of the most powerful sensing devices for various scientific and technological applications. From the viewpoint of bioscientific and biomedical applications, an image sensor plays an important role in imaging technologies such as microscopy and endoscopy.

In most applications, image sensors have been used with an optical component that projects a remote target image onto a pixel array implemented on the image sensor. However, in some applications, image sensors or line sensors are used without projection optics. Taking this on-chip sensing configuration, we can expect novel bioscientific sensing technologies. We can extend the functionality of the sensor not only within light sensing but also into sensing technologies for other measurement targets, such as electric, electrochemical, and pH images.

Since both an optical image sensor and on-chip electric sensor are fabricated using CMOS technology, it is possible to implement both optical sensing and electric sensing functionalities in an on-chip CMOS sensor. However, although there are a large number of reports on various on-chip biosensor technologies, there are a few reports on multifunctional implementations [1,2,3,4,5,6,7,8,9,10,11]. This paper reviews the concept of a combined optical + electric multifunctional implementation using CMOS image sensors for on-chip bioscientific applications.

## 2. Application Targets for Multifunctional On-Chip Image Sensors and Approaches to Multifunctional Implementation

The use of image sensors in on-chip configurations is a reasonable approach to realize a compact, portable, and low-cost measurement system for bioscientific measurement targets such as living cells, DNA, and proteins. The basic idea of on-chip optical bioscientific imaging using a charge coupled device (CCD) was reported in the early 1990s [12,13], and the concept was applied to a CMOS image sensor in the 2000s [14,15,16,17,18].

In addition, another group of large-scale integration (LSI)-based on-chip sensors using electric measurement schemes has been continuously developed. In most part of the CMOS LSI structure, metal wiring layers are buried in thin passivation layers, and it is easy to realize electric coupling between on-chip measurement target and electric circuitry in both capacitive and conductive manners. Direct electric (potential/current) sensing technologies to detect extracellular activities [19,20] or DNA [21], and electrochemical imaging technologies [22,23,24,25], are expected to be implemented using CMOS on-chip sensors. The idea of monolithic integration of CMOS circuitry onto electrodes for neural sensing was also proposed in the 1980s [26] and has seen continuous development [27,28]. Moreover, a bare Si surface or one covered with a thin layer of a dielectric material such as SiO_2_ or SiN_x_ exposed to the measurement target can be realized with slight modification of the standard CMOS fabrication processes. This structural feature means that conventional CMOS is natively compatible with not only electric sensing schemes that use the metal wiring layer as the sensing electrode, but also implementation of ion-sensitive field-effect transistor (ISFET)-based biosensing technologies [29,30,31,32,33,34,35,36,37,38,39]. The implementation of ISFET using CMOS devices was proposed and demonstrated in the 1980s [40,41], and compatibility with currently available standard CMOS fabrication technologies has also been demonstrated [42,43,44,45,46].

For an optical + electric multifunctional implementation, the active pixel sensor (APS) design [47] is the only realistic light-sensing architecture, and is used in most current CMOS image sensors. Only secondary variations around the photodiode structure are available in APS pixel circuitry. The photodiode structure used in conventional APS circuitry (3-transitor APS) can be replaced with a photogate structure. Alternatively, in some fabrication technologies, a pinned photodiode [48] is available for higher performance (4-transistor APS).

In addition, variations in electric sensing architecture are available for implementation of multifunctional CMOS image sensors. To minimize electric perturbation of the measurement target, potential sensing can be achieved using the gate of a MOS transistor as the sensing input [49,50,51,52,53,54,55,56,57,58,59,60]. With this “passive” configuration, only a small amount of capacitive charge transfer occurs between the sensing input and measurement target, and no net current flows between them. Both capacitive [49,50,51,52,53,54,55,56,57,58,59] and conductive [60] couplings are possible in this configuration, and ISFET can be included in this measurement scheme. Since these measurement schemes can be realized with circuits as simple as APS, the potential for high-resolution on-chip imaging exists with this approach.

**Figure 1:**
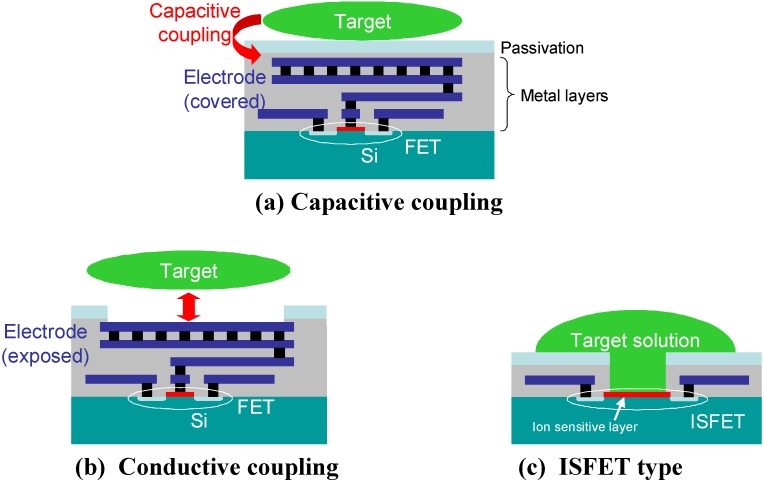
Variations of “passive” electric sensing configurations using the gate of MOS as sensing input. (**a**) Capacitive coupling; (**b**) Conductive coupling; and (**c**) ISFET type.

Figure 1 illustrates the concept of on-chip “passive” electric sensing using the gate of a MOS transistor as the sensing input.

The proposed multifunctional CMOS image sensor with electric sensing functionality using a passive (gate-input) configuration is described in Section 3.

The passive sensing configuration using a gate input is advantageous in minimizing electric perturbation and achieving high resolution. However, we cannot apply a voltage or inject current using the passive configuration. A sensing electrode connected via switching circuitry is required to realize “active” measurement schemes; that is, to apply voltage or inject current from or to the sensing electrode [61,62,63,64,65,66,67,68,69,70,71,72,73,74]. In the active sensing approach, we can achieve both capacitive coupling (in which the sensing electrode is covered with a dielectric insulation layer) and conductive coupling (with exposed sensing electrodes).

With capacitive coupling through an insulated electrode, we can apply a voltage or sense the on-chip potential capacitively. The advantage of the active electric sensing scheme is that on-chip potential profile can be controlled with no net current flow. By implementing such covered electrodes with the capability of applying a voltage, Guerrieri *et al.* realized a multifunctional sensor capable of controlling the positions of living cells cultured on a CMOS image sensor [9,10,11].

Alternatively, an intelligent multielectrode array (MEA) device can be realized by exposing the sensing electrode to detect the extracellular potentials of neural cells [61,62,63,64,65]. Conventional high-density electrochemical measurements are possible. We can perform electrochemical DNA detection or immunoassay [66,67,68,69,70,71,72,73,74] using a CMOS-based MEA-type sensor device.

Although such a CMOS-based electric sensor device would attract considerable interest, there are few reports on multifunctional implementation with an optical image sensor. However, we have developed a multifunctional CMOS image sensor with this type of active electric sensing functionality, as will be described in Section 4, Section 5 and Section 6.

## 3. Optical + Passive Electric Sensing CMOS Image Sensor

CMOS devices fabricated with standard CMOS technology have potential compatibility with on-chip capacitively coupled electric sensing. In standard CMOS technologies, metal wiring layers formed on the Si wafer are covered with an insulating layer for passivation and isolation. We can design a metal sensing electrode using the top metal layer, and the passivation layer formed in the standard fabrication process can be used as the dielectric insulating layer for capacitively coupled electric sensing.

Since there is no current flow from the pixel to the measurement target, the electric sensing pixel can be designed without a mechanism for current conveyance. The readout circuit used for a conventional CMOS image sensor pixel (3-transistor active pixel sensor, or 3-Tr APS) can be used to read out the electric potential of the sensing electrode.

**Figure 2:**
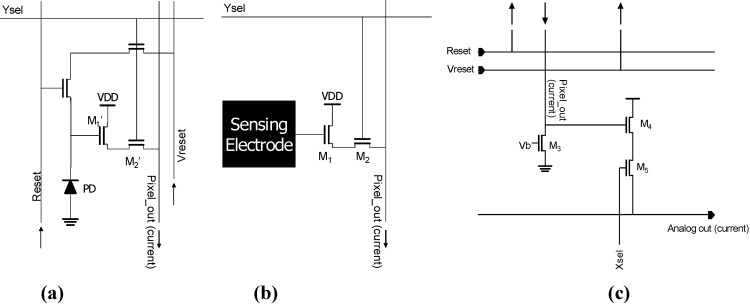
Schematic diagrams of **(a)** an optical sensing pixel (APS) and **(b)** a passive electric sensing pixel (capacitive coupling) with **(c)** columnar readout circuitry (adapted from [1]).

Figure 2 shows schematic diagrams of (a) an optical sensing pixel (APS) and (b) passive electric sensing pixel (capacitive coupling) with (c) columnar readout circuitry [1]. As seen in Figure 2, the circuits for the optical sensing pixel and electric sensing pixel are quite similar. The circuit of the passive electric sensing pixel is even simpler than that of the optical sensing pixel. The readout of the electric sensing pixel is realized with source follower circuitry, as for the optical sensing pixel.

When the Ysel input of the pixel is pulled up to high level, the readout transistor M_1_ (or M_1_’) is connected to the column readout line. A current source transistor M_3_ is implemented in the columnar readout circuitry (Figure 2(c)). The connection between the readout transistor M_1_ (or M_1_’) of the selected pixel and current source M_3_ in the columnar circuit is established using a vertical readout line. Using this temporarily configured source follower readout circuitry, the voltage of the photodiode (PD) node (or sensing electrode) can be transferred to the columnar readout circuit.

**Figure 3:**
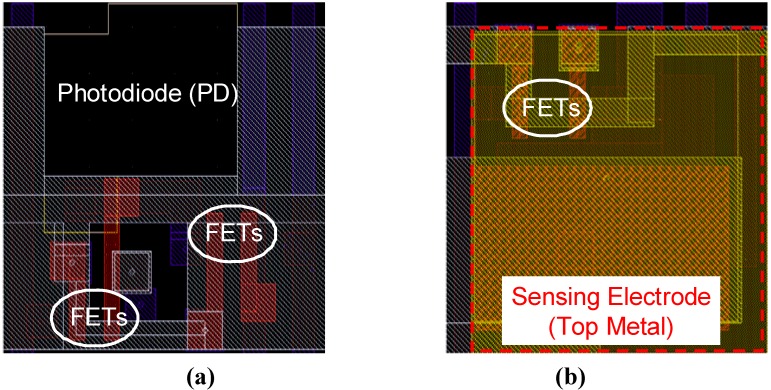
Layouts of the **(a)** optical sensing pixel and **(b)** passive electric sensing pixel (capacitive coupling) (adapted from [1]).

Figure 3 shows layouts of the (a) optical sensing pixel and (b) passive electric sensing pixel (capacitive coupling) [1]. We used 0.35 µm 2-poly, 4-metal standard CMOS technology for this design. The pixel dimensions were 7.5 µm × 7.5 µm. Not only the pixel size but also the alignments of the vertical/horizontal lines are designed to be same for the two kinds of pixels. Since these two pixels are interchangeable, we can design an optical + passive electric dual-image CMOS sensor by only replacing some of the pixels of the CMOS image sensor.

**Figure 4:**
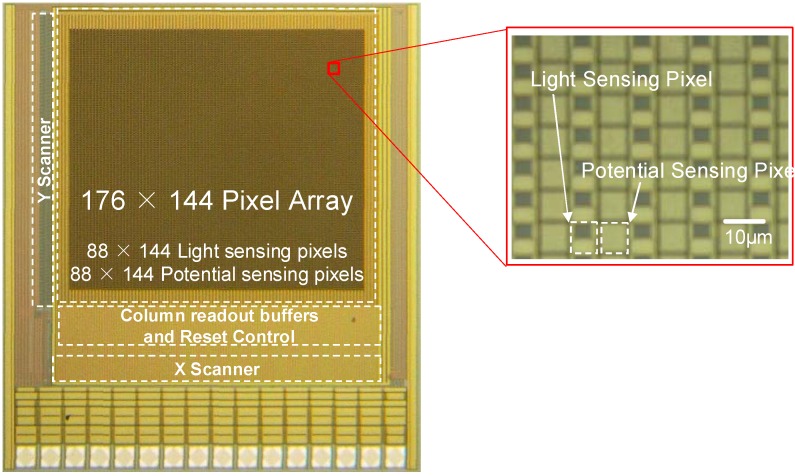
Layout of an optical + passive electric dual-image CMOS sensor (adapted from [1] © 2006 Elsevier Science).

Figure 4 shows the layout of an optical + electric dual-image CMOS sensor [1]. The sensor has a 320 × 240 pixel array consisting of alternating columns of optical sensing pixels and electric sensing pixels. The operational voltage of the sensor is 3.3 V and the signal is read out as analog voltage.

**Figure 5:**
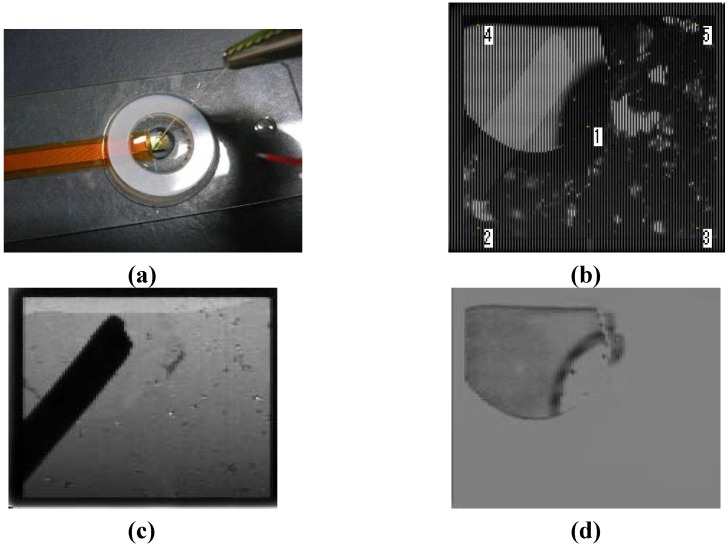
**(a)** Experimental setup; **(b)** as-captured image; **(c)** optical image; and **(d)** electric image captured by the optical and electric dual-image CMOS sensor (adapted from [1]).

Figure 5 shows images captured by the optical and electric dual-image CMOS sensor [1]. Figure 5 (a) is the experimental setup, Figure 5 (b) is the as-captured image, and Figure 5 (c) and Figure 5 (d) are optical and electric images, respectively. The as-captured image (Figure 5 (b)) is a combined image consisting of alternating columns showing distributions of the light intensity and electric potential. We can separate the as-captured image into optical (Figure 5 (c)) and electric (Figure 5 (d)) images.

**Figure 6:**
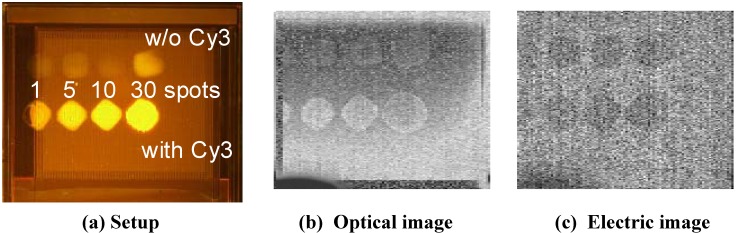
Images of on-chip DNA spots captured with and without illumination (adapted from [3]). (**a**) The setup; (**b**) Optical image; and (**c**) Electric image, of DNA spots with and without Cy3 modification.

As a functional demonstration of the optical + passive electric dual-image CMOS sensor, we performed imaging of on-chip DNA spots. We applied spots of 20-mer oligonucleotide directly on the sensor chip and captured an image of the spots. Details of the experimental materials and protocols have been previously reported [3]. Figure 6 shows captured images with and without light illumination. We find two rows of DNA spots on the images: the lower row consists of DNA spots modified with Cy3, and the upper row consists of spots without modification with any fluorophore. In the optical image, the spots were observed only under illumination. By contrast, the spots in electric images are observed regardless of illumination. Differing from the case for the spots in the illuminated image, the difference in the contrast between the spots with and without Cy3 modification is very small in the electric images.

The results suggest that we can sense the DNA spots in two ways. In general, in DNA microarray technology, the target DNA with unknown sequences is modified with a fluorophore, such as Cy3 or Cy5, and hybridized with preformed DNA spots on the microarray chip. In the optical image (Figure 6 (b)), we can observe the amount of fluorophore included in the spot, which is usually used to determine the amount of target DNA adhering to the probe DNA spots. However, the electric images provide information on the total amount of the DNA spots (Figure 6 (c)). Using these two values, not only the amount of adhered species but also the amount of probe DNA lost in the DNA hybridization process can be estimated.

As another possible application, the sensor can be used as an *in vitro* neural observation device. As reported by many groups [61,62,63,64,65], on-chip electric imaging is a promising technology for *in vitro* neural imaging technology. Using the present sensor, we can capture real-time optical and electric images of neural activity. Sawada *et al.* have been working on multifunctional image sensors with electric sensing functionality based on ISFET-type configuration [6,7,8] (see Figure 1 (c)). They developed optical + pH multifunctional image sensors. They designed both the light- and pH-sensing pixels based on charge transfer gate structure. The gate functions as a photogate in a light-sensing pixel and as a proton-sensitive ISFET in a pH-sensing pixel. The proton sensitivity inherent to the SiN_x_ passivation layer was advantageously used to realize pH imaging. They successfully demonstrated the optical + pH dual imaging. Due to the compatibility of light-sensing and pH-sensing pixel, they have to realize also dual imaging using same sensing area in the pixel [8].

## 4. Optical + Active Electric Sensing CMOS Image Senor

The optical and passive electric image sensors described in the previous section have a functional feature of on-chip passive electric sensing with capacitive coupling. We have developed another optical and electric dual-image CMOS sensor designed for on-chip active electric measurements [4]. To establish conductive coupling for the on-chip measurement target, the sensing electrode must be exposed.

**Figure 7:**
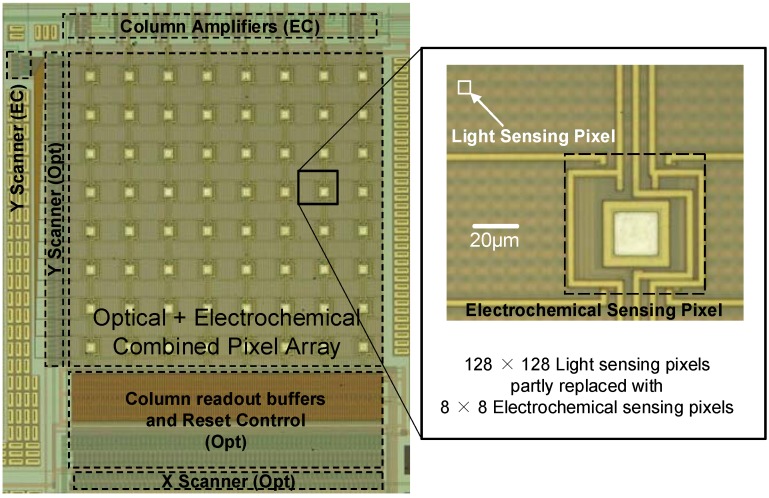
Layout of the optical and active electric (conductive coupling) dual-image CMOS sensor (adapted from [4]).

Figure 7 shows the layout of the optical and active electric dual-image CMOS sensor [4]. We used 0.35 µm 2-poly, 4-metal standard CMOS technology for the sensor described in the previous section. The base structure of the sensor is a 128 × 128 CMOS optical image sensor. The design of the optical sensing pixel is identical to that of the sensor in the previous section. In the pixel array, 8 × 8 conductively coupled electric sensing electrodes with a size of 30.5 µm × 30.5 µm are implemented.

**Figure 8:**
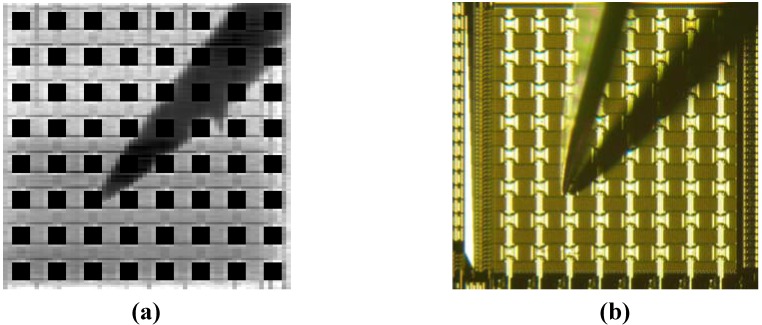
**(a)** Optical image captured with the dual-image CMOS sensor and **(b)** experimental situation (adapted from [4]).

Figure 8 shows a typical optical image captured with the dual-image CMOS sensor [4]. We can see a clear image of the shadow of the probe electrode. The image has 64 square data-less voids corresponding to the sensing electrode array.

**Figure 9:**
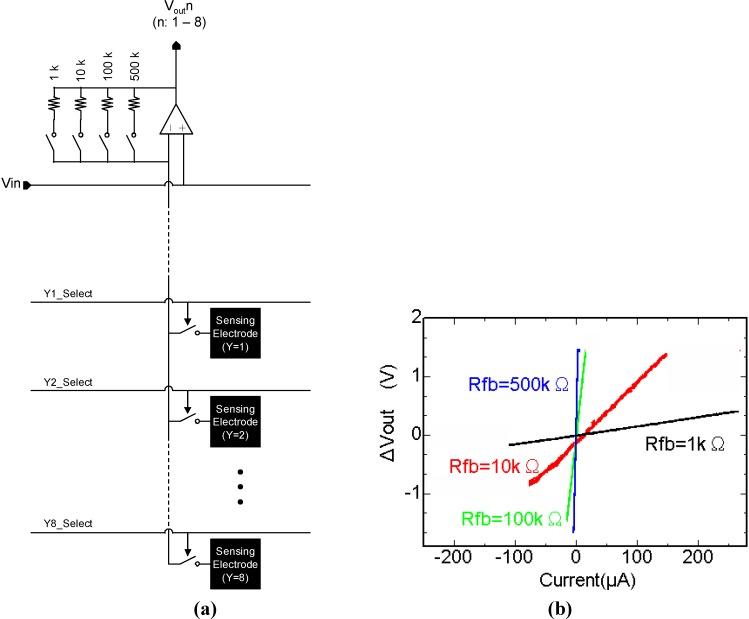
**(a)** Block diagram of the conductively coupled electric sensing pixel array and **(b)** measurement characteristics of the potentiometric amplifier unit (adapted from [4]).

Figure 9 (a) is a block diagram of the conductively coupled electric sensing pixel array [4]. Eight sensing electrodes in each column are connected to a columnar signal line. The column signal lines are connected to a column sensing amplifier. As the column sensing amplifiers, voltage follower circuits are configured using a two-stage amplifier. We can control the voltage of the column amplifier and sense the current flow from the sensing electrode into the on-chip sensing target. Thus, the column amplifier can be used as a column-parallel on-chip potentiometer. The feedback resistance in the voltage follower can be selected to obtain appropriate current sensitivity.

**Figure 10:**
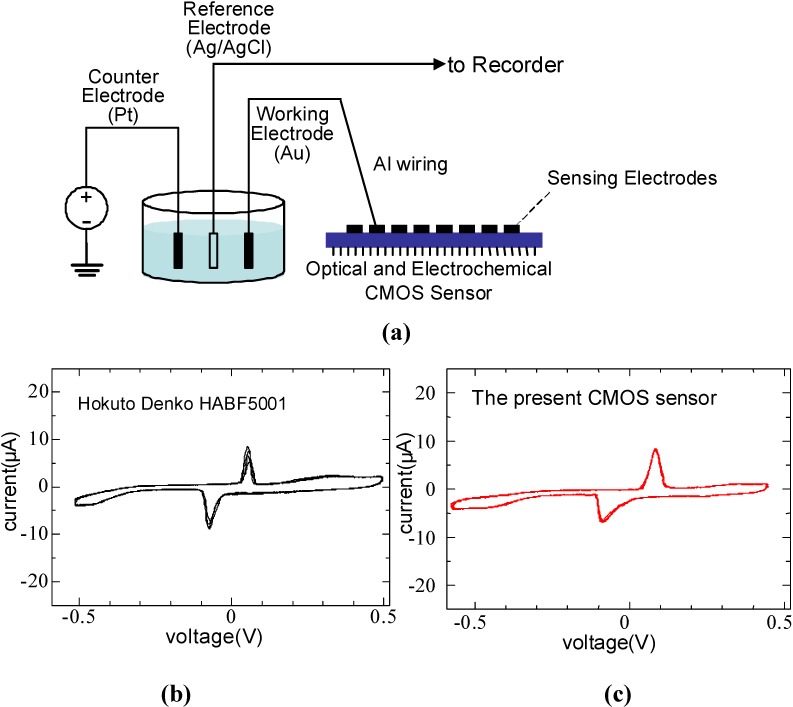
**(a)** Measurement setup; and C-V curves measured with **(b)** a conventional potentiostat and **(c)** the present CMOS sensor (adapted from [4]).

Figure 9 (b) shows the measurement characteristics of the potentiometric amplifier unit [4]. The sensing terminal (inverting input of the column amplifier unit) was maintained at 1.5 V, which is applied as the non-inverting input Vin. The current injected into the sensing terminal was controlled by an external current source. The four lines in Figure 10 show ÄVout = Vout − Vin as functions of the input current. Figure 10 shows that the sensor can be used in the current range approximately between 10 nA and 100 µA.

To confirm the functionality of the electrochemical sensor, we performed off-chip C-V measurements [4]. Figure 10 (a) shows the measurement configuration. An Au working electrode with a diameter of 1 mm was connected to the sensing terminal of the electrochemical sensing amplifier unit. The potential of the Au working electrode was maintained at 1.5 V, applied as Vin. The three-electrode configuration was used in the off-chip C-V measurement. A Pt wire electrode was used as a counter-electrode, and an Al/AgCl electrode was used as a reference electrode. The potential of the counter-electrode was scanned in the range of 1.0–2.0 V. For comparison, C-V curves were also measured for the same Au working electrode with a commercially available potentiostat (Hokuto Denko HABF5001). Figure 10 (b) and Figure 10 (c) show C-V curves obtained in the off-chip experiment. The small difference between the C-V curves measured with the electrochemical sensing amplifier unit of the present sensor (Figure 10 (b)) and a conventional potentiostat (Figure 10 (c)) shows that the electrochemical sensing amplifier unit correctly works as a voltage-controlled current-measurement circuit.

In designing the two-dimensional, voltage-controlled current-sensing device, the access scheme for electric sensing pixels should be chosen from two options: column-parallel access or random access. In this work, we implemented column-parallel, voltage-controlled current-sensing functionality as the active electric sensing method. The column-parallel access scheme is advantageous in obtaining two-dimensional imaging, but local stimulation or local voltage application/current injection is not possible. In addition, the column-parallel access scheme offers another option in the readout: parallel readout or scanned readout. When the number of pixels in the electric sensing array is small, the parallel readout scheme is possible, as shown in Figure 9 (a). However, for a large pixel number, the parallel readout is not realistic.

In Section 5, we describe another optical + electric CMOS image sensor that is capable of both passive and active electric sensing. We also implemented a scanned readout scheme for the electric sensing functionality. In Section 6, a multifunctional CMOS image sensor for local current injection is described. We employed a random-access scheme for electric sensing pixel.

Guerrieri *et al.* developed another type of optical + active electric multifunctional sensor [9,10,11]. Actually, their sensor employed active electric pixels only for voltage application. They realized two-dimensional voltage application using capacitively coupled active electric pixels. The two-dimensional potential distribution was used to control the positions of cultured cells by dielectrophoresis [9,10,11]. The optical imaging functionality was used to determine the positions of cells cultured on the sensor chip. A 320 × 320 array consisting of multifunctional pixels was implemented on the sensor. The pixels were equipped with conventional APS circuitry for light sensing, and single bit memory to connect each capacitively coupled pixel electrode to either a high or low voltage. The two-dimensional potential pattern was formed by storing the single bit of control data on all of the pixels. Column and row decoders were implemented to scan the pixels for both programming the voltage-application and light-sensing functionalities.

## 5. Pixel Level Integration of Passive and Active Electric Sensing Functionality

In this section, we describe an optical and electric dual-sensing pixel and columnar circuitry to achieve optical/potential/voltammetric triple imaging functions.

**Figure 11:**
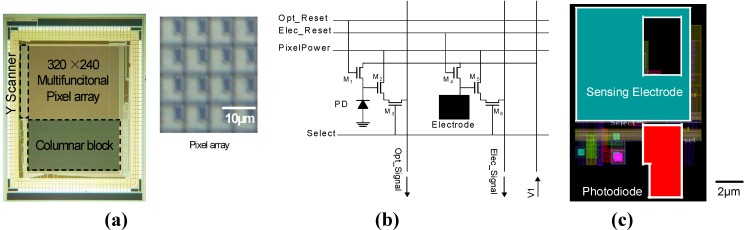
**(a)** Full layout, **(b)** pixel schematics, and **(c)** pixel layout of the optical and passive/active electric dual-sensing pixel designed for the multifunctional CMOS image sensor (adapted from [5]).

We designed the sensor with 0.35 µm 2-poly, 4-metal standard CMOS technology [5]. Figure 11 shows (a) the full layout, (b) the pixel schematics, and (c) the layout of the optical and electric dual-sensing pixels designed for the multifunctional CMOS image sensor. The dimensions of a pixel are 7.5 µm × 7.5 µm. A pixel consists of a PD, a sensing electrode and reset (M1, M4), an amplifier (M2, M5), and select (M3, M6) transistors for the PD and electrode. Since the pixel was designed with parallel output configuration, the optical and electric sensing functions can be simultaneously operated. The PD size is 10.8 µm^2^. The circuitry of the optical sensing part of the pixel is a standard 3-Tr APS, as for the previous sensors.

In the electric sensing part of the pixel, the sensing node (cathode) of the PD in the APS is replaced with the sensing electrode. The sensing electrode with a size of 38 µm^2^ is formed with the top metal layer. As fabricated, the sensing electrode is covered with passivation layers of the standard CMOS technology. As fabricated, the sensor is used as a passive electric image sensor.

The sensing electrode has a reset transistor (M4). Without the reset transistor for the electrode, the potential sensing function suffers from residual charge in the electrodes as a large offset in the pixel characteristic. The reset line for the electrode is also used to establish a current path in the active electric imaging function. To use the active electric sensing functionality, the top passivation layer should be removed to expose the sensing electrode.

**Figure 12:**
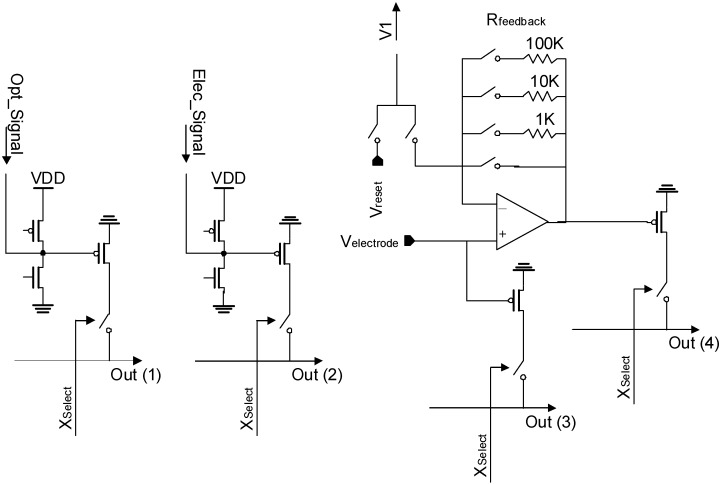
Schematic diagram of columnar circuitry (adapted from [5] ).

Figure 12 is a schematic diagram of the columnar circuitry of the sensor [5]. The columnar circuitry consists of two sets of columnar load units and a pmos source follower for optical and electric columnar signal lines, respectively. Each load unit has both nmos and pmos loads. Switching the voltage applied to a pixel (PixelPower in Figure 11 (a)) and biasing pmos and nmos loads appropriately, the pixel amplifier transistor (M2, M5) can be operated in both source follower and common source mode.

## 6. Implementation of Random-Access Functionality onto Active Electric Pixel for Local Stimulation or Current Injection

The optical and electric multifunctional CMOS image sensor gives rise to another promising technological possibility of two-dimensional integration of a light source on a CMOS array sensor. In the last decade, owing to breakthroughs in optogenetics, light has become a medium not only of observation but also of stimulation of the biological system.

**Figure 13:**
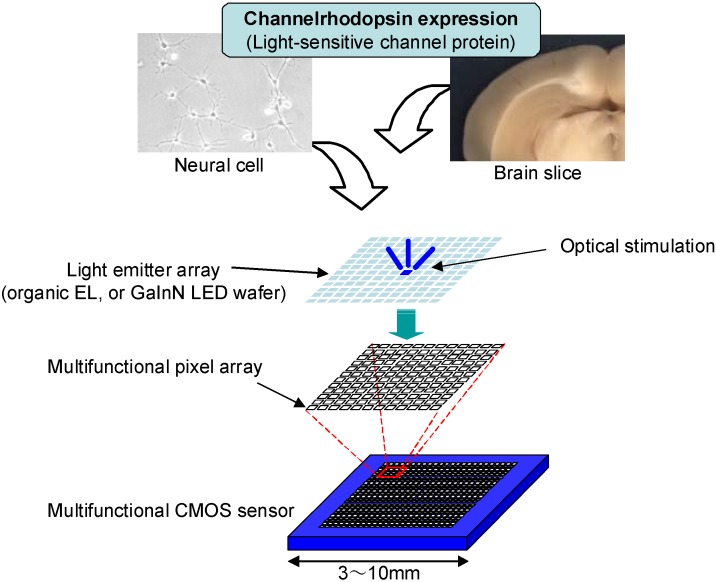
Concept of the multifunctional biointerface (neural-interface) device.

A slight modification of the sensor design allows the application of two-dimensionally random-access current injection. Figure 13 shows the concept of the multifunctional biointerface (neural-interface) device with a target field of application of brain/neural science. Currently, we are designing a CMOS multifunctional sensor with addressable current injection functionality. A light-emitter array such as an organic electroluminescence (EL) or light-emitting diode array will be directly formed on or bonded onto the sensor surface.

**Figure 14:**
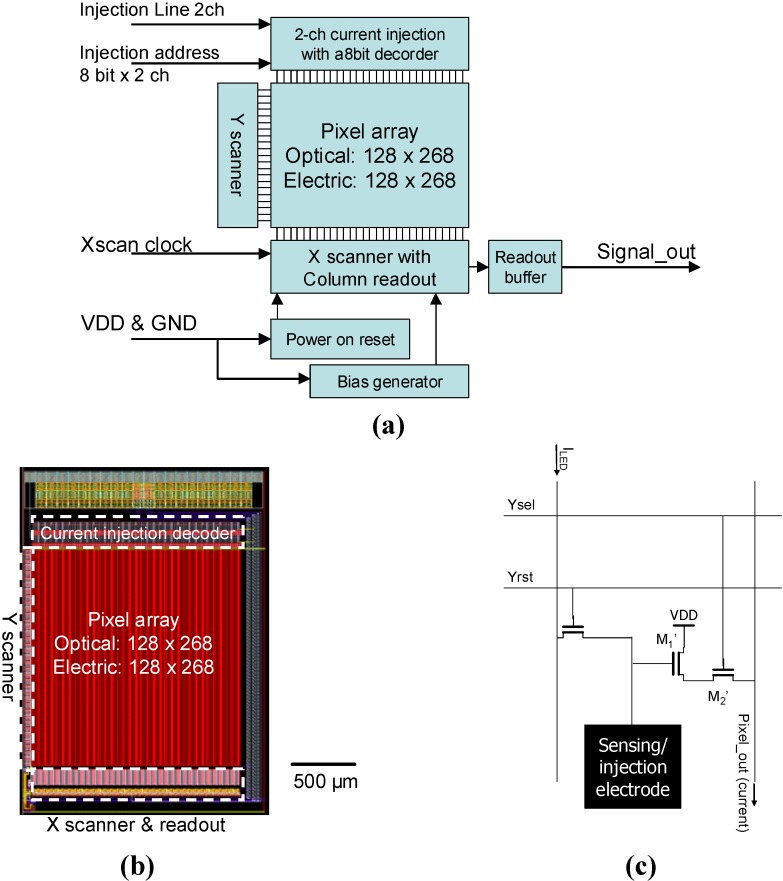
**(a)** Block diagram, **(b)** layout, and **(c)** pixel schematics of the sensor with addressable injection.

Figure 14 shows a block diagram and layout of the sensor with random-access injection functionality. For this sensor, we use functionally separated pixels, as was for the sensor described in Section 3. We add current injection capability through a direct input line that can be selectively connected to one of the columnar current supply lines. In combination with a vertical scanner originally implemented for the functionality of a CMOS image sensor, we can select one electric pixel for current injection.

**Figure 15:**
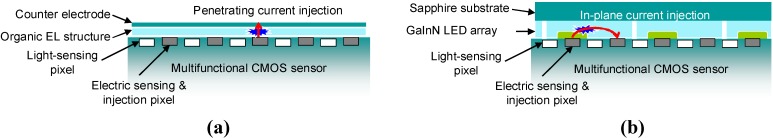
Current paths in the integrated light source with **(a)** an organic electroluminescence (EL) layer and **(b)** a GaInN LED array.

Figure 15 shows the possible current paths that will be realized in the integration of a light source array. Two current injection lines with a seven-bit decoder are implemented to establish current paths. Two injection lines can be connected to two of the 128 electric pixel columns. The column current supply lines are connected to the array electrodes when Yrst is high, as for the optical sensing pixels.

When we choose one of the electric pixel rows, we can perform penetrating (single-pixel) current injection that is compatible with the light-emission structure driven with current through the layers, such as organic EL layers (see Figure 15 (a)). We can also perform in-plane (inter-pixel) current injection using two current injection lines simultaneously. This injection scheme is compatible with a light-emission structure with horizontal current flow, such as a GaInN light-emitting diode array (see Figure 15 (b)).

Currently, the CMOS chip is under fabrication. The implementation of a light-emitter array and performance characterization will be reported in the future.

## 7. Conclusions

We developed multifunctional CMOS image sensors with target applications of on-chip biosensing/bioimaging. Passive and active electric sensing pixels for on-chip sensing with capacitive and conductive couplings compatible with CMOS image sensor architecture were proposed and designed. Several types of the optical + electric CMOS on-chip image sensors are demonstrated. On the basis of this technology, we also propose a new type of CMOS on-chip biosensor with an integrated on-chip light-emitter array.

## References

[1] Tokuda T., Yamamoto A., Kagawa K., Nunoshita M., Ohta J. (2006). A CMOS image sensor with optical and potential dual imaging function for on-chip bioscientific applications. Sens. Actuators A.

[2] Tokuda T., Ng D., Yamamoto A., Kagawa K., Nunoshita M., Ohta J. An optical and potential dual-image CMOS sensor for on-chip neural and DNA imaging applications. Proceedings of Technical Digest of IEEE International Symposium on Circuits and Systems.

[3] Tokuda T., Kadowaki I., Kagawa K., Nunoshita M., Ohta J. (2007). A new scheme for imaging on-chip dry DNA spots using optical/potential dual-image complementary metal oxide semiconductor sensor. Jpn. J. Appl. Phys..

[4] Tokuda T., Tanaka K., Matsuo M., Kagawa K., Nunoshita M., Ohta J. (2007). Optical and electrochemical dual-image CMOS sensor for on-chip biomolecular sensing applications. Sens. Actuators A.

[5] Tokuda T., Kadowaki I., Kagawa K., Nunoshita M., Ohta J. An optical/potential/voltammetric multifunctional CMOS image sensor for on-chip biomolecular/neural sensing applications. Proceedings of International Conference on Solid State Devices and Materials.

[6] Sawada K., Ohshina T., Hizawa T., Takao H., Ishida M. (2005). A novel fused sensor for photo-and ion-sensing. Sens. Actuators B.

[7] Nakazawa H., Ishida M., Sawada K. (2010). Progressive-type fused pH and optical image sensor. Jpn. J. Appl. Phys..

[8] Nakazawa H., Ishii H., Ishida M., Sawada K. (2010). A fused pH and fluorescence sensor using the same sensing area. Appl. Phys. Express.

[9] Manaresi N., Romani A., Medoro G., Altomare L., Leonardi A., Tartagni M., Guerrieri R. (2003). A CMOS chip for individual cell manipulation and detection. IEEE J. Solid-State Circuits.

[10] Medoro G., Manaresi N., Leonardi A., Altomare L., Tartagni M., Guerrieri R. (2003). A lab-on-a-chip for cell detection and manipulation. IEEE Sensors J..

[11] Romani A., Manaresi N., Marzocchi L., Medoro G., Leonardi A., Altomare L., Tartagni M., Guerrieri R. Capacitive sensor array for localization of bioparticles in CMOS lab-on-a-chip. Proceeding of 2004 IEEE International Solid-State Circuits Conference (IEEE Cat. No.04CH37519).

[12] Lamture J.B., LBeattie K., Burke B.E., Eggers M.D., Ehrlich D.J., Fowler R., Hollis M.A., Kosicki B.B., Reich R.K., Smith S.R., Varma R.S., Hogan M.E. (1994). Direct detection of nucleic acid hybridization on the surface of a charge coupled device. Nucleic Acids Res..

[13] Eggers M., Hogan M., Reich R.K., Lamture J., Ehrlich D., Hollis M., Kosicki B., Powdrill T., Beattie K., Smith S. (1994). A microchip for quantitative detection of molecules utilizing luminescent and radioisotope reporter groups. BioTechniques.

[14] Eltoukhy H., Salama K., El Gamal A., Ronaghi M., Davis R. A 0.18 µm CMOS 10^−6^ lux bioluminescence detection system-on-chip. Proceeding of 2004 IEEE International. Solid-State Circuits Conference.

[15] Salama K., Eltoukhy H., Hassibi A., El Gamal A. (2004). Modeling and simulation of luminescence detection platforms. Biosens. Bioelectron..

[16] Mallard F., Marchand G., Ginot F., Campagnolo R. (2005). Opto-electronic DNA chip: High performance chip reading with an all-electric interface. Biosens. Bioelectron..

[17] Abshire P.A. CMOS contact imager for monitoring cultured cells. Proceeding of 2005 IEEE International Symposium on Circuits and Systems.

[18] Eltoukhy H., Salama K. (2006). A 0.18-µm CMOS bioluminescence detection lab-on-Chip. IEEE J. Solid-State Circuits.

[19] Pine J. (1980). Recording action potentials from cultured neurons with extracellular microcircuit electrodes. J. Neurosci. Methods.

[20] GROSS G., Rieske E., Kreutzberg G., Meyer A. (1977). A new fixed-array multi-microelectrode system designed for long-term monitoring of extracellular single unit neuronal activity *in vitro*. Neurosci. Lett..

[21] Hashimoto K., Ito K., Ishimori Y. (1994). Novel DNA sensor for electrochemical gene detection. Anal. Chim. Acta.

[22] Tanaka H., Yoshinobu T., Iwasaki H. (1999). Application of the chemical imaging sensor to electrophysiological measurement of a neural cell. Sens. Actuators B.

[23] Iwasaki Y., Tobita T., Kurihara K., Horiuchi T., Suzuki K., Niwa O. (2002). Imaging of electrochemical enzyme sensor on gold electrode using surface plasmon resonance. Biosens. Bioelectron..

[24] Hayashi K., Horiuchi T., Kurita R., Torimitsu K., Niwa O. (2000). Real-time electrochemical imaging using an individually addressable multi-channel electrode. Biosens. Bioelectron..

[25] Barker A.L., Unwin P.R., Gardner J.W., Rieley H. (2004). A multi-electrode probe for parallel imaging in scanning electrochemical microscopy. Electrochem. Commun..

[26] Najafi K., Wise K.D. (1986). An implantable multielectrode array with on-chip signal processing. IEEE J. Solid-State Circuits.

[27] Olsson R.H., Wise K.D. (2005). A three-dimensional neural recording microsystem with implantable data compression circuitry. IEEE J. Solid-State Circuits.

[28] Normann R.A. (2007). Technology insight: Future neuroprosthetic therapies for disorders of the nervous system. Nat. Clin. Pract. Neurol..

[29] Bergveld P. (1970). Development of an ion-sensitive solid-state device for neurophysiological measurements. IEEE Trans. Biomed. Eng..

[30] Bergveld P. (1991). A critical evaluation of direct electrical protein detection methods. Biosens. Bioelectron..

[31] Bergveld P. (2003). Thirty years of ISFETOLOGY What happened in the past 30 years and what may happen in the next 30 years. Sens. Actuators B.

[32] Berggren C., Stålhandske P., Brundell J., Johansson G. (1999). A feasibility study of a capacitive biosensor for direct detection of DNA hybridization. Electroanalysis.

[33] Berggren C., Bjarnason B., Johansson G. (2001). Capacitive biosensors. Electroanalysis.

[34] Martinoia S., Rosso N., Grattarola M., Lorenzelli L., Margesin B., Zen M. (2001). Development of ISFET array-based microsystems for bioelectrochemical measurements of cell populations. Biosens. Bioelectron..

[35] Bockelmann U., Pouthas F., Gentil C., Co D. (2004). DNA detection on transistor arrays following mutation-specific enzymatic amplification. Appl. Phys. Lett..

[36] Uslu F., Ingebrandt S., Mayer D., Böcker-Meffert S., Odenthal M., Offenhäusser A. (2004). Labelfree fully electronic nucleic acid detection system based on a field-effect transistor device. Biosens. Bioelectron..

[37] Sakata T., Miyahara Y. (2005). Detection of DNA recognition events using multi-well field effect devices. Biosens. Bioelectron..

[38] Sakata T., Miyahara Y. (2007). Direct transduction of allele-specific primer extension into electrical signal using genetic field effect transistor. Biosens. Bioelectron..

[39] Kim D.-S., Jeong Y.-T., Park H.-J., Shin J.-K., Choi P., Lee J.-H., Lim G. (2004). An FET-type charge sensor for highly sensitive detection of DNA sequence. Biosens. Bioelectron..

[40] Wong H.S., White M.H. A self-contained CMOS integrated pH sensor. Proceeding of 1988 Electron Devices Meeting.

[41] Bousse L., Shott J., Meindl J. (2002). A process for the combined fabrication of ion sensors and CMOS circuits. IEEE Electron Device Lett..

[42] Bausells J., Carrabina J., Errachid A., Merlos A. (1999). Ion-sensitive field-effect transistors fabricated in a commercial CMOS technology. Sens. Actuators B.

[43] Chin Y.L., Chou J.C., Sun T.P., Chung W.Y., Hsiung S.K. (2001). A novel pH sensitive ISFET with on chip temperature sensing using CMOS standard process. Sens. Actuators B.

[44] Hammond P., Ali D. (2004). Design of a single-chip pH sensor using a conventional 0.6-μm CMOS process. IEEE Sensors J..

[45] Hammond P.A., Cumming D.R.S. (2005). Performance and system-on-chip integration of an unmodified CMOS ISFET. Sens. Actuators.

[46] Milgrew M.J., Riehle M.O., Cumming D.R.S. (2005). A large transistor-based sensor array chip for direct extracellular imaging. Sens. Actuators.

[47] Fossum E.R. (1997). CMOS image sensors: Electronic camera-on-a-chip. IEEE Tran. Electron Devices.

[48] Teranishi N., Kohono A., Ishihara Y., Oda E., Arai K. No image lag photodiode structure in the interline CCD image sensor. Proceedings of 1982 International Electron Devices Meeting.

[49] Pancrazio J.J., Bey P.P., Loloee A., Manne S., Chao H.C., Howard L.L., Gosney W.M., Borkholder D.A., Kovacs G.T., Manos P., Cuttino D.S., Stenger D.A. (1998). Description and demonstration of a CMOS amplifier-based-system with measurement and stimulation capability for bioelectrical signal transduction. Biosens. Bioelectron..

[50] Gilchrist K.H., Barker V.N., Fletcher L.E., DeBusschere B.D., Ghanouni P., Giovangrandi L., Kovacs G.T. (2001). General purpose, field-portable cell-based biosensor platform. Biosens. Bioelectron..

[51] DeBusschere B.D., Kovacs G.T. (2001). Portable cell-based biosensor system using integrated CMOS cell-cartridges. Biosens. Bioelectron..

[52] Zeck G., Fromherz P. (2001). Noninvasive neuroelectronic interfacing with synaptically connected snail neurons immobilized on a semiconductor chip. Proc. Natl. Acad. Sci. USA.

[53] Eversmann B., Jenkner M., Hofmann F., Paulus C., Brederlow R., Holzapfl B., Fromherz P., Merz M., Brenner M., Schreiter M., Gabl R., Plehnert K., Steinhauser M., Eckstein G., Schmitt-Landsiedel D., Thewes R. (2003). A 128 × 128 CMOS biosensor array for extracellular recording of neural activity. IEEE J. Solid-State Circuits.

[54] Kovacs G.T. (2003). A electronic sensors with living cellular components. Proc. IEEE..

[55] Fromherz P. (2003). Semiconductor chips with ion channels, nerve cells and brain. Physica E.

[56] Lambacher A., Jenkner M., Merz M., Eversmann B., Kaul R.A., Hofmann F., Thewes R., Fromherz P. (2004). Electrical imaging of neuronal activity by multi-transistor-array (MTA) recording at 7 µm resolution. Appl. Phys. A.

[57] Meyburg S., Goryll M., Moers J., Ingebrandt S., Böcker-Meffert S., Lüth H., Offenhäusser A. (2006). N-Channel field-effect transistors with floating gates for extracellular recordings. Biosens. Bioelectron..

[58] Meyburg S., Stockmann R., Offenh A., Ingebrandt S. (2007). Advanced CMOS process for floating gate field-effect transistors in bioelectronic applications. Sens. Actuators.

[59] Fromherz P. (2008). Joining microelectronics and microionics: Nerve cells and brain tissue on semiconductor chips. Solid-State Electron..

[60] Berdondini L., van Der Wal P.D., Guenat O., de Rooij N.F., Koudelka-Hep M., Seitz P., Kaufmann R., Metzler P., Blanc N., Rohr S. (2005). High-density electrode array for imaging in vitro electrophysiological activity. Biosens. Bioelectron..

[61] Heer F., Franks W., McKay I., Taschini S., Hierlemann A., Baltes H. CMOS microelectrode array for extracellular stimulation and recording of electrogenic cells. Proceedings of 2004 IEEE International Symposium on Circuits and Systems (IEEE Cat. No.04CH37512).

[62] Heer F., Hafizovic S., Franks W., Ugniwenko T., Blau A., Ziegler C., Hierlemann A. CMOS microelectrode array for bidirectional interaction with neuronal networks. Proceedings of 2005 European Solid-State Circuits Conference.

[63] Hafizovic S., Heer F., Ugniwenko T., Frey U., Blau A., Ziegler C., Hierlemann A. (2007). A CMOS-based microelectrode array for interaction with neuronal cultures. J. Neurosci. Methods.

[64] Frey U., Egert U., Heer F., Hafizovic S., Hierlemann A. (2009). Microelectronic system for high-resolution mapping of extracellular electric fields applied to brain slices. Biosens. Bioelectron..

[65] Frey U., Sedivy J., Heer F., Pedron R., Ballini M., Mueller J., Bakkum D., Hafizovic S., Faraci F.D., Greve F., Kirstein K.-U., Hierlemann A. (2010). Switch-matrix-based high-density microelectrode array in CMOS technology. IEEE J. Solid-State Circuits.

[66] Li J., Xue M., Lu Z., Zhang Z., Feng C., Chan M. (2003). A high-density conduction-based micro-DNA identification array fabricated with a CMOS compatible process. IEEE Tans. Electron Devices.

[67] Schienle M., Paulus C., Frey A., Hofmann F., Holzapfl B., Schindler-Bauer P., Thewes R. (2004). A fully electronic DNA sensor with 128 positions and in-pixel A/D conversion. IEEE J. Solid-State Circuits.

[68] Dill K., Montgomery D.D., Ghindilis A.L., Schwarzkopf K.R., Ragsdale S.R., Oleinikov A.V. (2004). Immunoassays based on electrochemical detection using microelectrode arrays. Biosens. Bioelectron..

[69] Dill K., Montgomery D.D., Ghindilis A.L., Schwarzkopf K.R. (2004). Immunoassays and sequence-specific DNA detection on a microchip using enzyme amplified electrochemical detection. J. Biochem. Bioph. Methods.

[70] Frey A., Schienle M., Paulus C., Hofmann F., Schindler-Bauer P., Holzapfl B., Atzesberger M., Beer G., Fritz M., Haneder T., Hanke H., Thewes R. A digital CMOS DNA chip. Proceedings of 2005 IEEE International Symposium on Circuits and Systems.

[71] Stagni C., Esposti D., Guiducci C., Paulus C., Schienle M., Augustyniak M., Zuccheri G., Samori B., Benini L., Ricco B., Thewes R. Fully electronic CMOS DNA detection array based on capacitance measurement with on-chip analog-to-digital conversion. Proceedings of 2006 IEEE International Solid State Circuits Conference—Digest of Technical Papers.

[72] Gemma N., O’uchi S., Funaki H., Okada J., Hongo S. CMOS integrated DNA chip for quantitative DNA analysis. Proceedings of 2006 IEEE International Solid State Circuits Conference—Digest of Technical Papers, IEEE.

[73] Levine P.M., Gong P., Levicky R., Shepard K.L. (2008). Active CMOS sensor array for electrochemical biomolecular detection. IEEE J. Solid-State Circuits.

[74] Heer F., Keller M., Yu G., Janata J., Josowicz M., Hierlemann A. CMOS electro-chemical DNA-detection array with on-chip ADC. Proceedings of 2008 IEEE International Solid-State Circuits Conference.

